# Notes on Medical Matters and Medical Men in Paris

**Published:** 1847-10

**Authors:** 


*Notes on Medical Matters and Medical Men in Paris.* By David W. Yandell, M. D., of Louisville, Ky.

I take great pleasure in transmitting to you the following letter from Dr. Brooks, the young physician whom I have more than once mentioned as being engaged here in the study of Veterinary medicine. I am sure it will be read with interest. The Agricultural Society of Massachusetts is fortunate in having secured the services of an individual capable of devoting himself to the science with so much ardor as Dr. Brooks has manifested in the subject, and every merciful man will respond heartily to the wish so well expressed at the close of this letter, that the profession which holds a respectable position in all other civilized countries may soon find a place among the reputable avocations of our own. It cannot but strike one as strange that the diseases of the horse, an animal so much admired by Americans, and so necessary to the comfort and efficiency of our race, should have been so little studied. The odium which has hitherto been inseparable in our country from the term *horse doctor* will cease when the subject has enlisted such advocates as the author of this letter. I bespeak for it the careful perusal of the readers of the Journal.

My Dear Yandell :

The Veterinary science, after many an age of the most unjust and inconceivable neglect, has at last received from the French Government, a large portion of the favour and honour to which it is entitled. Especial colleges have been established at Lyons, Toulouse, and Alfort, in the northern, central and southern points of the kingdom, whence numbers of intelligent young men, thoroughly grounded in the principles and practice of their profession, and imbued with those sentiments of self-respect and independence which true learning and unquestioned value never fail to impart, are yearly sent forth into society to fulfil, conscientiously, their honorable and important missions.

The "Horse Doctor," in the English sense of the term, has happily ceased to exist,—and if the medicine of man has not as yet extended a cordial hand to her younger sister, it is that *La Veterinaire* has hardly had time to remove the enormous prejudices which ignorance and habit have accumulated against her, and that a certain feeling of hereditary dislike still lingers between the members of two sister sciences.

But the day is at hand, I hope, when the medicine and surgery of domestic animals shall be united, as in times of old, to that of humanity. The great principles of the healing art never change—their application alone varies. There should be then but one *type* of medical schools, where the universal laws of science being imparted to all alike, their various subdivisions may by natural aid be perfected.

*Claude Bourgelat* is a name dear, for a thousand reasons, to the veterinary science. He alone foresaw its future utility and glory—the inestimable advantages which it is destined to render, and now renders, to medicine, agriculture, philanthrophy—and only he was gifted with the energy and learning requisite to triumph over the numberless obstacles which obstructed his path. The labours of Bourgelat are almost incredible—naturally fond of the horse, he abandoned, for the sake of this noble animal, a career in which he had just made his debut with the greatest credit, and gave himself wholly up to the study of his qualities, mechanism, and diseases. He revised the works of his predecessors, and exposed their many errors, and he has left to posterity some of the most remarkable treatises, upon nearly every branch of veterinary medicine, which have ever appeared. His opinion is still an authority, and even his faults bear the stamp of his honesty and of his genius. The name of *Claude Bourgelat, the Founder of the Veterinary Colleges of France*, is as familiar to the ears of the French students as are their own names, and his writings adorn the shelves of every well-selected library.

But I almost forgot that I had promised to give you an account of the Royal College of Alfort. I will henceforth spare you all general reflections, and confine myself to my subject.

*Alfort* is, as you know, a little village situated upon the road to Lyons, about six miles from Paris. The veterinary school, to which it is indebted for its celebrity, contains upon an average some two hundred and fifty students, of whom about seventy-five enter annually, after having been previously subjected to a general, and, I think, insufficient examination. Diplomas, authorizing the title of Veterinary Physician, are conferred upon forty-five or fifty; of this number nearly one-half are destined for the army—entering the several cavalry regiments with the title of *Under Veterinary Assistants*, and with a rank corresponding to that of quartermaster.*

The duration of the studies is fixed at four years; examinations taking place at the close of each scholastic year, to decide whether or not the student is fitted to pass into a superior class, and to enter upon its more difficult pursuits. Such as fail to pass a satisfactory examination are obliged to recommence the studies of the year

* Marechal des logis.

which has elapsed, and those that fail twice in the same examination,* are dismissed as wanting in capacity. As these periodical tests are pretty severe, there is always a certain number of *veterans* (as the backsliders are called) in the inferior classes. A student may thus, *a la rigeur*, be compelled to pass eight consecutive years in the college, and may even then fail to succeed in his final examination, and leave without a diploma.

Chemistry and physics, in their application to veterinary medicine, botany, pharmacy, anatomy, general and special therapeutics, surgery, and hygiene are the principal branches of science taught at Alfort.

M. Euguene Renault, the Director of the institution, lectures upon Veterinary Jurisprudence, an intimate knowledge of which is all important to the practitioner.†

The hospitals are spacious and well ventilated; they are capable of receiving nearly one hundred patients, at the moderate rate of fifty cents a day each for boarding, medical advice and surgical operations being entirely gratuitous.

Operations are performed either in the open air, or in the amphitheatre, which is lighted from above, and in other respects adapted to the purpose. Their various stages, and the accidents liable to attend them, are thus advantageously seen by a large number of students at a time.

Diseases of the feet, as in the vicinity of all large cities, are very frequent at Alfort; and I think that—thanks to the admirable skill of M. Henri Bouley‡—their nature and treatment are better understood here than elsewhere.

Each member of the superior classes has a patient entrusted to his care, for which he is held responsible, and for the state of which he is periodically called upon to account. There is a stable especially destined for incurable subjects (*chevaux d'experience*), and within still another, situated at the extremity of the park, are confined glandered horses, and such as are affected with contagious diseases.

Vivisections, on an extensive scale, are performed by the students during the summer months; sixteen horses being sacrificed for this object weekly. Upon each animal are performed the capital and minor operations of veterinary surgery, such as lithotomy, extirpation of the fibro-cartilages of the ears, ligature of the carotid and other arteries, all operations on the feet, venesection in various parts of the body, extraction of the teeth,§ etc. Eight students operate in turn upon each subject, when this is practicable; thus,

* Except in rare instances.

† I am about to give in a paper intended for a northern Journal, a sketch of the laws now in operation in France touching the question of *soundness*.

‡ It is to be hoped that this gentleman will ere long publish, what is so much needed, a work upon Practical Veterinary Surgery.

§ This cruel operation is not, I believe, enjoined by the regulations, but I have seen students extract molar teeth, *en passant*, for their own satisfaction.

about one hundred operations, of more or less importance, are performed upon each beast ; as you may well imagine life rarely lingers to the last. I have no opinion to offer here upon this system, authorized by the French Government, of torturing animals to death, by way of recompense for the services which they may have previously rendered it. The sight is far from being a pleasant one, and its influence upon the minds of the pupils cannot, to say the least, be of a very elevating nature. Are the advantages of the practice sufficient to counterbalance its cruelties ? Have we a right to cause one creature to suffer beyond measure, for the benefit of others of its kind—or for our own benefit ? The intention of the vivisections is in part to habituate the student to the sight of blood, and to teach him to to be ever upon his guard against unexpected efforts of his patient.

The museum contains some very interesting objects, that I have not space here to enumerate. The newly elected Professor of Anatomy, M. Gonbaux, will seize, I am sure, every opportunity of adding to its already valuable collection of anatomical and pathological preparations.

The dormitories are in a separate building, which also contains the refectory and kitchens. Each chamber is occupied by six young men. The bed-steads are of iron, (and here I may venture to suggest that it is to be regretted that their use is not more general with us than it is, as well for public schools as in hospitals). Each of its occupants is in turn bound to wash out and keep clean the common room.

There is a botanical garden attached to the institution, in one portion of which the most useful medical plants are cultivated, and in another, those only which are employed in Veterinary Pharmacy.

The dog-kennels are next to the garden; they are sufficiently extensive and pretty well managed. The patients are kept under shelter during the winter season, and in summer they are chained during the day time to the walls of the yard, where they receive the benefit of the air and sun. Affections of the skin, eyes and ears, tetanus, the dystemper and hydrophobia, are among the disorders which I have oftenest observed among the dogs at Alfort. A series of very interesting and curious experiments were instituted last year, with the view of testing the contagion of this latter disease from the dog to other animals. I have seen it repeatedly produced in the horse and the sheep. There are few subjects more important and more interesting than this, and it is probable that we shall have before long from the able pen of M. Renault, the most perfect and learned treatise upon hydrophobia (or better still, *rage*) which has ever yet appeared.

There is always a *pig-stye* to be seen at the college, but it is rarely occupied by the animals for which it is intended. It is quite deserted at the moment in which I write. No very serious attention is paid to the maladies of the pig although they are theoretically professed in the course of the session. The last occupants of the stye were of English race crossed with that of China. M. Viborg, the directer of the Veterinary College of Copenhagen, has written an excellent treatise upon the diseases of this *edible* animal.

In their first year the students at the College at Alfort, are taught to forge, as also to form and apply shoes of various models to the foot of the horse. The forges are constructed upon simple and effective principles. There are six double furnaces at which the students work two at a time, in alphabetical order.

The dissecting rooms are in recently erected buildings, of which certain portions are yet unfinished. They consist of two spacious halls lighted from either side, and communicating with each other through the cabinet of the professor who, thus, is constantly close at hand with his scalpel and advice. For convenience of transport, the subjects are placed upon solid iron carriages, of a peculiar and convenient form.

The laboratory is to be removed at an early period to a new room expressly arranged for it. Another large building for the storing of fodder is in the course of construction.

A professor of horsemanship was formerly attached to the college, but for some reason or other the study of this accomplishment, so useful, if not essential, to the veterinary physician, above all if he be destined for the army, has been suppressed.

The drawing master has also disappeared. This, too, is to be regretted, for the art of drawing is of inestimable service to the anatomist and pathologist.

In this short letter upon the college of Alfort, I have not pretended to broach any of the numerous questions which at present agitate the veterinary world. I have perhaps written at once too little and too much, but should this superficial review fail to satisfy your interest and curiosity concerning the science, I may attempt, upon some future occasion, to do higher justice to so important a subject.

The Veterinary Colleges of France are the most perfect that exist; let us take them for our model, and seek to emancipate in the new world a noble science, that still struggles for independence and justice in the land of our fathers.

Your friend, EDWARD BROOKS, Jr.

ALFORT, June 8th, 1847. [*Western Jour. of Med. and Surg.*

